# Antibiotic Killing of Diversely Generated Populations of Nonreplicating Bacteria

**DOI:** 10.1128/AAC.02360-18

**Published:** 2019-06-24

**Authors:** Ingrid C. McCall, Nilang Shah, Adithi Govindan, Fernando Baquero, Bruce R. Levin

**Affiliations:** a Department of Biology, Emory University, Atlanta, Georgia, USA; b Department of Microbiology, Ramón y Cajal Institute for Health Research (IRYCIS), Ramón y Cajal University Hospital, CIBERESP, Madrid, Spain; c Antibiotic Resistance Center, Emory University, Atlanta, Georgia, USA

**Keywords:** antibiotic stasis, antibiotic-killing, nonreplicating bacteria, persisters, stationary phase

## Abstract

Nonreplicating bacteria are known to be (or at least commonly thought to be) refractory to antibiotics to which they are genetically susceptible. Here, we explore the sensitivity to killing by bactericidal antibiotics of three classes of nonreplicating populations of planktonic bacteria: (i) stationary phase, when the concentration of resources and/or nutrients are too low to allow for population growth; (ii) persisters, minority subpopulations of susceptible bacteria surviving exposure to bactericidal antibiotics; and (iii) antibiotic-static cells, bacteria exposed to antibiotics that prevent their replication but kill them slowly if at all, the so-called bacteriostatic drugs. Using experimental populations of Staphylococcus aureus Newman and Escherichia coli K-12 (MG1655) and, respectively, nine and seven different bactericidal antibiotics, we estimated the rates at which these drugs kill these different types of nonreplicating bacteria. In contrast to the common belief that bacteria that are nonreplicating are refractory to antibiotic-mediated killing, all three types of nonreplicating populations of these Gram-positive and Gram-negative bacteria are consistently killed by aminoglycosides and the peptide antibiotics daptomycin and colistin, respectively. This result indicates that nonreplicating cells, irrespectively of why they do not replicate, have an almost identical response to bactericidal antibiotics. We discuss the implications of these results to our understanding of the mechanisms of action of antibiotics and the possibility of adding a short-course of aminoglycosides or peptide antibiotics to conventional therapy of bacterial infections.

## Abstract (precis)

Nonreplicating bacteria are known to be (or at least commonly thought to be) refractory to antibiotics to which they are genetically susceptible. Here, we explore the sensitivity to killing by bactericidal antibiotics of three classes of nonreplicating populations of planktonic bacteria: (i) stationary phase, when the concentration of resources and/or nutrients are too low to allow for population growth; (ii) persisters, minority subpopulations of susceptible bacteria surviving exposure to bactericidal antibiotics; and (iii) antibiotic-static cells, bacteria exposed to antibiotics that prevent their replication but kill them slowly if at all, the so-called bacteriostatic drugs.

## INTRODUCTION

For therapeutic purposes, the relationship between the concentrations of antibiotics and the rates of growth and death of bacteria, i.e., the pharmacodynamics, is almost exclusively studied *in vitro* under conditions that are optimal for the action of these drugs: relatively low densities of bacteria growing exponentially in media and under culture conditions where all members of the exposed population have equal access to these drugs, resources, wastes, and metabolites excreted into the environment. To be sure, in some sites and tissues in acutely infected hosts, relatively low densities of the target pathogens may be growing exponentially at their maximum rate and thus are under conditions that are optimal for the action of antibiotics. However, this situation is almost certainly uncommon in established, symptomatic, and thereby treated infections where the offending bacteria are likely to be compartmentalized in different sites and tissues and confronting the host’s immune defenses (1).

Infecting populations of bacteria may be nonreplicating for different reasons and by different mechanisms. First, they may have exhausted the locally available resources and thus modified their environment so their populations are at or near stationary phase (2–6). Second, although local nutrients may be sufficient for their replication, for hosts treated with bactericidal drugs these bacteria may be minority populations of physiologically refractory survivors, the so-called “persisters” (7–13). Third, the offending bacteria may be nonreplicating because of exposure to bacteriostatic antibiotics, a state we shall refer to as antibiotic-induced stasis. Fourth, infecting bacteria may be slowly replicating or at stationary phase inside phagocytes or other host cells (14, 15) or attached to the surfaces of tissues or prosthetic devices and within polysaccharide matrices know as biofilms (16) and thus not replicating for one or more of previously described reasons (4, 17).

The concept of antibiosis has been classically linked to the fight against actively replicating microbes invading host tissues. With some exceptions associated with permeability (18), the susceptibility of bacteria to killing by bactericidal antibiotics is related to their rate of replication. In fact, with beta-lactams, the rate at which bacteria are killed has been shown to be strictly proportional to the rate at which the population is growing (19, 20). The same trend seems to occur for other bactericidal agents, such as fluoroquinolones, aminoglycosides, glycopeptides, and lipopeptides (21–24). It is well known that exposure to bacteriostatic antibiotics markedly reduces the efficacy of beta-lactam drugs to kill bacteria (25–27). However, except for these cases of antagonism between bacteriostatic and bactericidal drugs and the classical studies by R. Eng et al. (28) in 1991, there is remarkably little information about the pharmacodynamics of antibiotics for nonreplicating populations of bacteria, despite the potential clinical implications.

In this investigation, we address two fundamental questions about the pharmacodynamics of nonreplicating bacteria. What antibiotics and to what extent do these drugs kill nonreplicating bacteria? With respect to their susceptibility to antibiotic-mediated killing, are bacteria in these different nonreplicating states physiologically similar? To address these questions, we compare the activity of antibiotics on nonreplicating bacterial populations obtained by different procedures. We present the results of experiments estimating the susceptibility of various nonreplicating populations of S. aureus and E. coli to killing by nine and seven different bactericidal antibiotics, respectively. We consider three types of nonreplicating states of planktonic bacteria: (i) those at stationary phase in oligotrophic culture; (ii) the nonreplicating survivors of exposure to bactericidal antibiotics, i.e., persisters; and (iii) bacteria that survive exposed to bacteriostatic antibiotics, i.e., antibiotic-static populations. In contrast to the popular conception that antibiotics are ineffective at killing nonreplicating bacteria (19, 29, 30), a number of existing bactericidal antibiotics, albeit not the beta-lactam drugs, can kill nonreplicating bacteria in all three states even when these antibiotics are administered at relatively low concentrations. The results of our experiments indicate that the same classes of antibiotics, aminoglycosides and peptides, are particularly effective at killing nonreplicating bacteria irrespective of the mechanism responsible for their failure to replicate. In addition to being relevant clinically, these results are interesting mechanistically since they suggest that nonreplicating bacteria of different types may share a cell physiology with respect to their interactions with antibiotics.

## RESULTS

### Antibiotic-mediated killing of exponentially growing bacteria.

**FIG 1:**
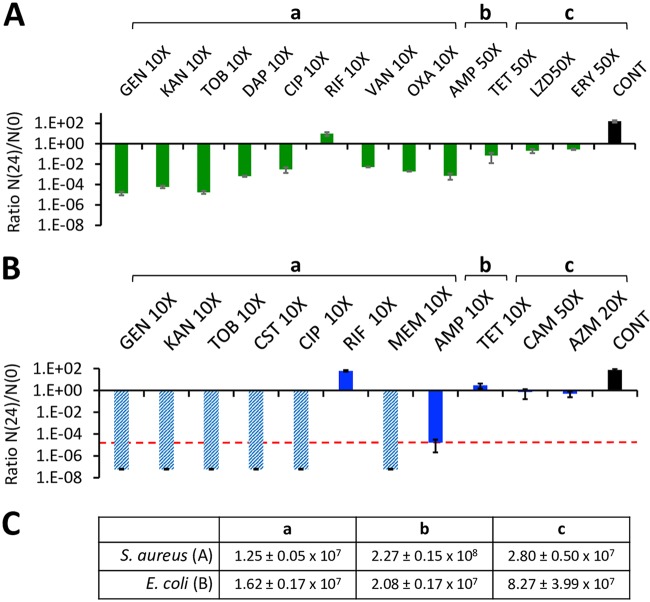
Antibiotic-mediated killing of exponentially growing cells. The ratios of viable 24-h to initial viable cell densities, N(24)/N(0), of exponentially growing broth cultures exposed to concentrations of bactericidal and bacteriostatic antibiotics (multiples of MIC values) were determined. (A) Staphylococcus aureus Newman in MHII; (B) Escherichia coli MG1655 in LB. The means and standard errors of the N(24)/N(0) ratios from three independent experiments each with three samples were determined. The broken line indicates the limit of detection (10^2^ cells per ml; hatched bars mean that this limit was surpassed in the assay). CONT is the control, i.e., bacteria growing in antibiotic-free medium. (C) The initial, N(0), densities of S. aureus and E. coli in the experiments discussed in panels A and B are shown in panel C.

As a baseline for our consideration of the antibiotic susceptibility of nonreplicating bacteria, we explore the response of exponentially growing populations S. aureus and *E coli* MG1655 to antibiotics. The results of these experiments are presented in Fig. 1.

For S. aureus, the aminoglycosides, gentamicin, kanamycin, and tobramycin kill to the greatest extent, with reductions in viable cell density of more than 4 orders of magnitude. Daptomycin, ciprofloxacin, vancomycin, oxacillin, and ampicillin are clearly bactericidal and reduce the viable cell density by 2 to 3 orders of magnitude. The increase in the N(24)/N(0) ratio for rifampin can be attributed to the ascent of rifampin-resistant mutants. Even at 50× MIC, tetracycline, linezolid, and erythromycin are effectively bacteriostatic. When exponentially growing cultures of E. coli are exposed to 10× MIC of gentamicin, kanamycin, tobramycin, colistin, ciprofloxacin, and meropenem, the viable cell density is below that which can be detected by plating. At 10× MIC, ampicillin reduces the viable cell density of exponentially growing E. coli by more than 4 orders of magnitude. As with S. aureus, the failure of rifampin to reduce the viable cell density can be attributed to the ascent of rifampin-resistant mutants.

### Antibiotic-mediated killing of stationary-phase bacteria.

For the stationary phase experiments with both S. aureus and E. coli, we used cultures that had been incubated under optimal growth conditions for 48 h. To estimate the amount of unconsumed, residual resources in these 48-h stationary-phase cultures, and thus the capacity for additional growth, we centrifuged and filtered (0.20-μm pore size) 48-h cultures of these bacteria. We then added, ∼10^3^ cells from overnight cultures to the cell-free filtrates and estimated the initial viable cell density and the viable cell density after 24 h of incubation, respectively, i.e., N(0) and N(24). The results of these experiments with four independent E. coli cultures and three independent S. aureus cultures suggest no significant growth for E. coli, with a N(24)/N(0) ratio of 0.36 ± 0.22, and some growth for S. aureus, with a N(24)/N(0) ratio of 10.91 ± 5.13. It should be noted, however, that the absence of growth or, in the case of S. aureus, the presence of limited growth in these spent media may be a reflection of the increase in the pH of this media, that is, from pH 7.0 at time zero to pH 8.5 at 48 h (31).

**FIG 2:**
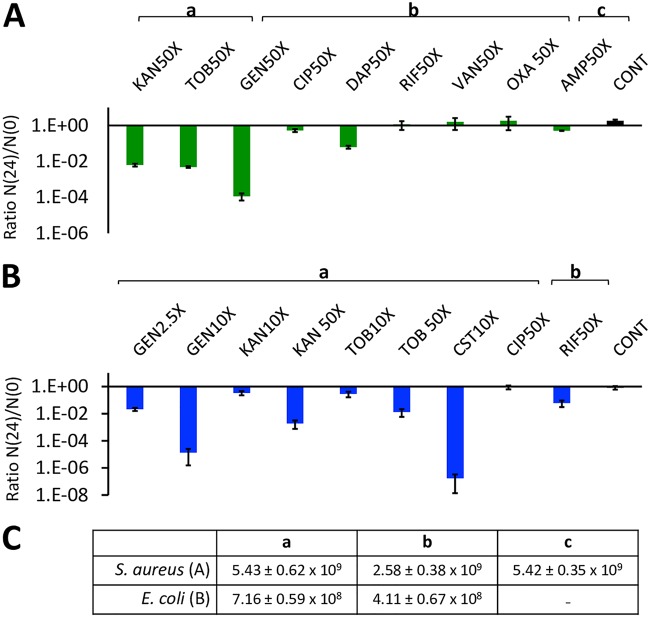
Antibiotic treatment of stationary-phase bacteria. The ratios of the viable cell densities after and before 24 h of exposure to bactericidal antibiotics, N(24)/N(0), were determined. Cultures of S. aureus (A) and E. coli (B) grown for 48 h were treated with the indicated MICs of different drugs and antibiotic-free controls (CONT). The means and standard errors of N(24)/N(0) ratios for 5 and 11 independent experiments for panels A and B, respectively, are shown. (C) The means and standard errors of the initial viable cell densities, N(0), for these experiments are shown in panel C.

At 48 h, the viable cell densities of the stationary-phase cultures were estimated (as shown in Materials and Methods). In Fig. 2, we present the results of this stationary-phase experiment. In the absence of treatment (the control), there is no significant mortality between 48 and 72 h for either S. aureus or E. coli. For S. aureus, only high concentrations of the aminoglycosides gentamicin, kanamycin, and tobramycin and the cyclic peptide daptomycin are effective in reducing the viable density of these 48-h stationary-phase cultures (Fig. 2A). For E. coli, the aminoglycosides gentamicin, tobramycin, and kanamycin are also effective for killing stationary-phase cells, as is colistin. There is no evidence that the other bactericidal antibiotics tested, i.e., ciprofloxacin and rifampin, killed stationary-phase E. coli.

### Antibiotic-mediated killing of *E. coli hipA7* and *S. aureus* persisters generated by exposure to bactericidal antibiotics (bactericidal persisters).

**FIG 3:**
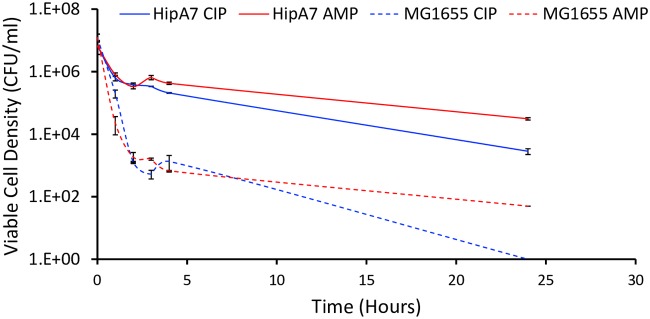
Dynamics of *hipA*7 persister formation. Changes in the viable cell density of exponentially growing E. coli MG1655 (broken lines) and an *hipA7* mutant of this strain (solid lines) exposed to 10× MIC ampicillin (red) and 10× MIC ciprofloxacin (blue) are shown. All data points represent the means and standard errors of three independent measurements. The average initial cell densities were (1.23 ± 0.34) × 10^7^ CFU/ml and (6.71 ± 3.16) × 10^6^ CFU/ml for MG1655 and *hipA7*, respectively.

In the case of E. coli, the number of viable cells surviving exposure to 10× MIC ampicillin and ciprofloxacin, that is, the persisters, was too low to test for the susceptibility of these bacteria to killing by other antibiotics. To address this issue, we restricted our E. coli persister experiments to *hipA7* (the Moyed mutant [32]), a construct that produces 10^3^ to 10^4^ times more persisters than does the wild type due to an increase in the basal level of (p)ppGpp synthesis (33). This is illustrated in Fig. 3, where we compare the dynamics of formation and the relative densities of persisters for E. coli MG1655 and the *hipA7* construct exposed to 10× MIC ampicillin and 10× MIC ciprofloxacin.

**FIG 4:**
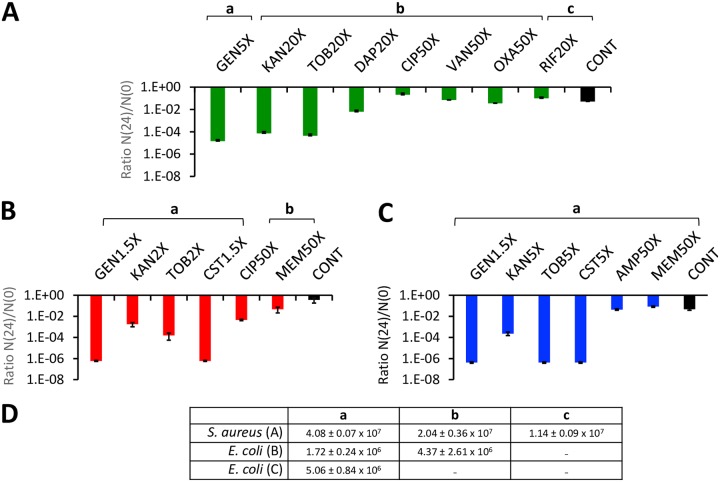
Antibiotic-mediated killing of bactericidal persisters. The ratios of viable cell densities of bactericidal persisters after and before 24 h of exposure to the noted concentrations of bactericidal antibiotic, N(24)/N(0), were determined. (A) S. aureus treated with 25× MIC ampicillin to generate the persister N(0); (B) E. coli *hipA7* treated with 10× MIC ampicillin to generate the persister N(0); (C) E. coli *hipA7* mutant treated with 10× MIC ciprofloxacin to generate the persister N(0). The means and standard errors of the N(24)/N(0) ratios for three independent experiments were determined. (D) The means and standard errors of the N(0) densities for these experiments are shown in panel D.

For S. aureus, we restricted our experiments to exploring the sensitivity of persisters to treatment with bactericidal antibiotics to ampicillin-generated persisters. In our pilot experiments generating these persisters with ciprofloxacin, kanamycin, and tobramycin, the density of the surviving cells, the persisters, was too low to test their susceptibility to killing by other antibiotics. To generate these S. aureus persisters, we used a protocol similar to a protocol used previously (34). The results of these experiments are presented in Fig. 4A. The extent to which these ampicillin-exposed S. aureus die following subsequent treatment is reflected by the N(24)/N(0) ratios of the controls (CONT) in Fig. 4. These S. aureus persisters are refractory to killing at 50× MICs of the bactericidal antibiotics ciprofloxacin, vancomycin, and oxacillin and 20× MIC rifampin. This is not the case for the aminoglycosides; 5× MIC gentamicin and 20× MIC tobramycin and kanamycin reduce the viable cell densities of these persisters by nearly 3 orders of magnitude. Albeit to an lower less than these aminoglycosides, at 20× MIC the cyclic peptide daptomycin also kills these ampicillin-generated S. aureus persisters.

The *hipA7* persisters were prepared by exposure to ampicillin and ciprofloxacin according to a protocol similar to that used by Keren et al. (35). The results of these experiments are presented in Fig. 4B and C. Relative to the controls, low, but super-MICs of the aminoglycosides gentamicin, kanamycin, and tobramycin and also the peptide colistin reduced the viable cell density of the E. coli *hipA*7 persisters by 3 to 6 orders of magnitude. Even at 50× MIC, the other antibiotics have little or no effect in reducing the viable cell density of the E. coli *hipA7* persisters.

### Antibiotic-mediated killing of antibiotic-induced static populations.

**FIG 5:**
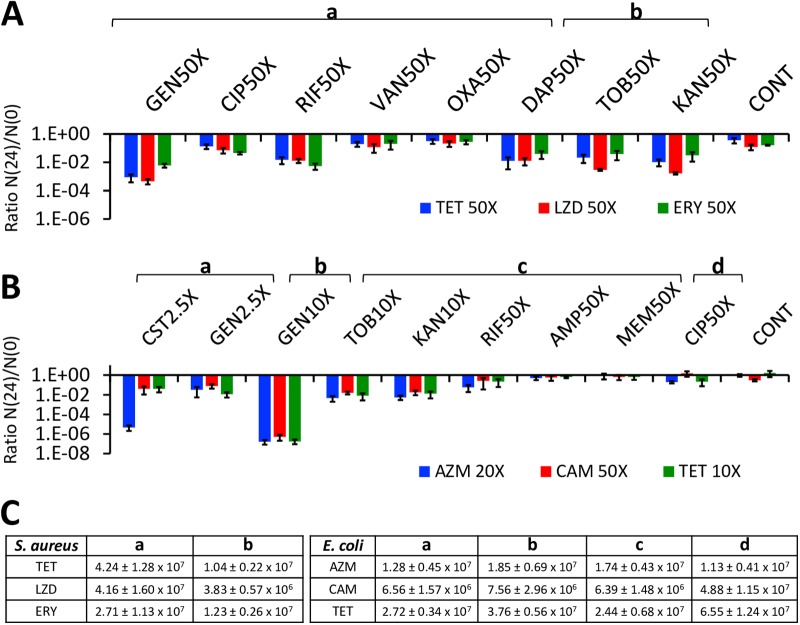
Antibiotic-mediated killing of antibiotic-static populations. The means and standard errors of the N(24)/N(0) ratios for S. aureus (A) and E. coli (B) were determined. The concentrations of the antibiotics as multiples of MICs used to generate the static populations and for subsequent treatment are noted in the figure. (C) The initial densities of S. aureus antibiotic-static populations receiving tetracycline (TET), linezolid (LZD), and erythromycin (ERY) treatments are shown in the S. aureus panel (left side of panel C). The initial densities of E. coli antibiotic-static populations receiving azithromycin (AZM), chloramphenicol (CAM), and tetracycline (TET) treatments are shown in the E. coli panel (right side of panel C).

Antibiotic-induced static populations were generated by exposing exponentially growing S. aureus and E. coli to bacteriostatic drugs for 24 h, followed by second antibiotics for another 24 h (as shown in Materials and Methods). The results of these experiments are presented in Fig. 5.

The S. aureus antibiotic-static populations surviving exposure to tetracycline, erythromycin, and linezolid are killed by the aminoglycosides, rifampin, and daptomycin. The E. coli antibiotic-static populations surviving exposure to azithromycin, chloramphenicol, and tetracycline are readily killed by the aminoglycosides and colistin and marginally if at all so by high concentrations of rifampin. Higher concentrations of gentamicin (10× MIC) are more effective than 10× MIC of the other aminoglycosides.

## DISCUSSION

During the course of an infection, several different conditions contribute to the nonreplicating status of initially growing populations of the infecting bacteria, triggering type I persisters, slow growers that are generated by exposure to a stress signal (36). Among these conditions are the stationary phase resulting from a dearth of the nutrients and resources in the infected tissues, the formation of biofilms, and the immune defenses, primarily engulfment by professional and amateur phagocytic host cells. Therapy with antibiotics will also result in the production of nonreplicating populations of bacteria, persisters surviving exposure to bactericidal antibiotics, and stasis induced by bacteriostatic drugs. We postulate that although they are generated in different ways, common mechanisms are responsible for the failure of these bacteria to replicate and thereby for the same classes of antibiotics to kill them. These mechanisms involve the general stringent response, a strongly regulated process governed by the alternative sigma factor RpoS (35) upregulated by the accumulation of hyperphosphorylated guanine nucleotides, as the “alarmone” (p)ppGpp (37–39). That regulation might in fact reflect the metabolism of persisters which is oriented toward energy production, with depleted metabolite fluxes, which perpetuates the slow growth (12, 40).

The contribution of the stringent response to antibiotic-induced stress is not well understood. A number of observations suggest that antibiotic exposure can trigger a RpoS stationary response and the generation of nonreplicating populations. Subinhibitory concentrations of beta-lactams induce the stringent response (41, 42). Response to stress by bacteriostatic antibiotics acting on the ribosome (as macrolides, chloramphenicol, or tetracyclines) is likely to be manifested by the reduction in protein synthesis, which might reduce the building up of (p)ppGpp, but that might be overcompensated for by reduced degradation of this nucleotide (43, 44). Proteome analysis of erythromycin-exposed “permeable” E. coli suggests a RpoS-regulated profile (45). In fact, subinhibitory concentrations of bacteriostatic antibiotics induce the stringent response, leading to beta-lactam tolerance (46).

Cationic peptides, including polymyxins, do not elicit an RpoS response but rather increase the permeability of the cell membranes and thereby act as rapid “external killers” (47, 48). Whether the aminoglycosides can elicit a RpoS response is unclear at this time. As is the case of other protein synthesis inhibitors, this machinery might be less effective in the presence of the antibiotic than in its absence; the same thing occurs with oxidative stress responses (44). The rapid killing observed in our work (see Fig. 1) may well be because the stringent response has no time to develop when exposed to these drugs. However, if aminoglycosides can induce the RpoS response, this might favor aminoglycoside transport into cells that results in membrane damage and killing of nongrowing cells (49).

The cellular immune response might also be responsible for generating nonreplicating populations. *Salmonella* RpoS-dependent genes are activated into the intracellular environment of eukaryotic cells (50). An RpoS-activated system contributes to survival of E. coli and *Salmonella* to the phagocyte oxidase-mediated stress resistance (51, 52) and influences the intracellular survival of *Legionella* in macrophages and in *Acanthamoeba* cells (53–55). Intracellular survival is also related to the overexpression of heat shock stress proteins, promoting nonreplication (56, 57).

These studies suggest that nonreplicating bacteria might also arise by the stringent response derived from professional and/or nonprofessional phagocytosis. Of course, aminoglycosides and peptide antibiotics do not enter in eukaryotic cells, but nonreplicating bacteria can be released from phagocytes lysed by bacterium-induced programmed necrosis, contributing to the chronification of the infection (58). Antibody-antibiotic conjugates might improve therapy of phagocytized bacteria where aminoglycosides and peptides are excluded (15).

We postulate that essentially the same set of cellular responses occurs in the different stress-inducing conditions that bacteria encounter during the infection process, and different classes of antibiotics are similarly effective in killing these different types of nonreplicating cells. These effects are probably are expressed as a hormetic (dose-dependent) stress response (59, 60). Of course, nongrowth is a complex mechanism in which the final antibiotic effects are influenced by those resulting from different coexisting stresses, for instance, nongrowth resulting from ribosome hibernation could facilitate some degree of gentamicin tolerance (61). The nongrowth phenotype is therefore causally complex, which negatively influences the full quantitative repeatability of the experiments.

It is well known that stationary phase populations of bacteria are refractory to killing by some antibiotics (19, 29, 30). The results presented here illustrate that this is not the case for other antibiotics, and in particular some aminoglycosides and peptide antimicrobials kill stationary-phase populations of E. coli and S. aureus. This observation is not new; it has been known for some time that the aminoglycosides and the cyclic peptides daptomycin and colistin can kill stationary-phase bacteria (24, 61–63). Less seems to be known about the susceptibility to killing by bactericidal antibiotics of the other nonreplicating states of bacteria considered here, the persisters and bacteria with antibiotic-induced stasis.

Although there have been many studies of persistence, relatively little is known about the susceptibility of these nonreplicating bacteria to antibiotics other than those used to generate them. One exception to this is a study by Allison et al. (64), who demonstrate that by adding metabolites, E. coli and S. aureus persisters in the form of biofilms become susceptible to killing by aminoglycosides. Our results with the ampicillin-generated planktonic S. aureus persisters are fully consistent with these observations. Results with the *hipA7* persisters used here also suggest that E. coli “natural” persisters are sensitive to killing by even low concentrations of the aminoglycosides and the peptide colistin.

There is also a relative dearth of quantitative information about the susceptibility to antibiotic-mediated killing of nonreplicating bacteria induced by exposure to bacteriostatic antibiotics. To be sure, within the first decade after the discovery of antibiotics, there was evidence that exposure of bacteria to bacteriostatic drugs reduces the efficacy of bactericidal to kill these bacteria (65), and these observations were corroborated more recently (27). Early observations concerning the lower efficacy of penicillin in static cells produced by chloramphenicol and the tetracyclines have engendered what some may see as an immutable law in the practice of antibiotic therapy: do not mix bacteriostatic and bactericidal drugs. However, we show that some existing bactericidal antibiotics are quite effective in killing bacteria that are not replicating because of their exposure to bacteriostatic antibiotics, what we refer to here as antibiotic-static populations.

In summary, stationary-phase S. aureus populations are killed at a substantial rate by the aminoglycosides and to a lesser extent by daptomycin. S. aureus persisters generated by exposure to ampicillin are also killed by the aminoglycosides and the lipopeptide daptomycin, but not the other drugs tested. Antibiotic-induced static populations of S. aureus maintain the same killing profile, with aminoglycosides and daptomycin as the sole killing agents, except, in this case, rifampin. Some aminoglycosides and the peptide colistin are also effective at killing stationary-phase E. coli, whereas the other antibiotics tested were not. This is also true for the *hipA7* E. coli persister and E. coli “suffering” from the stasis induced by ribosome-targeting bacteriostatic antibiotics. This homogeneous pattern of response to antibiotic killing in slow-growth populations from different origins supports a basically homogeneous physiology in all type I persisters.

### Potential clinical implications.

In recent years, there has been a great deal of interest in discovering and developing drugs to treat nonreplicating populations of bacteria, particularly those associated with biofilms. A prime example of this is the use by Lewis and coworkers (66) of a novel antibiotic, acyldepsipeptide (ADEP4). Despite growing efforts in the endeavor to find new antibiotics to kill nonreplicating bacteria, the results presented here suggest that existing antimicrobials may well be up to that task. A valid concern is that the antibiotics with this virtue are among the more toxic drugs, i.e., aminoglycosides and peptides (67–69). It should be noted, however, that relatively low and possibly nontoxic concentrations of the aminoglycosides and the peptide colistin can kill E. coli antibiotic-static and *hipA7* persisters. Most importantly, as has been the case with cancer chemotherapy, there are conditions under which some toxic side effects of systemic treatment are more than made up for by the sometimes life-saving benefits of that treatment (70). Of course, inhalation therapy, providing very high local concentrations of aminoglycosides or peptide antibiotics, has proven its efficacy in mostly nongrowing populations of Pseudomonas aeruginosa and Staphylococcus aureus involved in chronic lung colonization in cystic fibrosis patients (71). Also, high local concentrations of these antibiotics have been useful in intravesical therapy of recurrent urinary tract infections (72) or in catheter-locking solutions to treat catheter-related bloodstream infections (73).

How important persisters are clinically is, at this juncture, not all that clear. Persisters remain susceptible to phagocytosis and other elements of the innate immune system, the main factor influencing control of infections, can be attributed to the innate immune system, and they would play little or no role in reducing the efficacy of antibiotic therapy (74). Consistent with this yet-to-be-tested (but testable) hypothesis in experimental animals and patients is the observation that for immunocompetent patients, bacteriostatic drugs are as effective for treatment as highly bactericidal agents (75, 76).

As intriguing as they may be scientifically, planktonic persisters surviving exposure to bactericidal antibiotics are not the majority of the nonreplicating bacteria present during the infection process. Stationary-phase bacteria resulting from a local shortage of nutrients, nongrowing populations induced by bacteriostatic agents, biofilm populations, and phagocytosed bacteria (eventually released by the lysis of the engulfing phagocytes) are likely to be the majority of phenotypically antibiotic-resistant bacteria in an established infection. They are certainly involved in the chronification of infections, as well as subsequent reactivations and relapses. It may well be that, along with the standard therapy, the addition of a short-course administration of antibiotics, such as the aminoglycosides and peptides, that kill these nonreplicating bacteria may well accelerate the course of treatment and increase the likelihood of its success.

## MATERIALS AND METHODS

### Bacteria.

Staphylococcus aureus Newman was obtained from William Shafer, Emory University, E. coli K-12 MG1655 was obtained from Ole Skovgaard, Roskilde University, and the high-frequency persister strain of E. coli K-12 (*hipA7*) constructed by Moyed and Betrand (32) was obtained from Kyle Allison, Emory University.

### Liquid culture.

For the S. aureus Mueller-Hinton broth (MHII; catalog no. 275710 [BD]) and for E. coli lysogeny broth (LB; catalog no. 244620 [Difco]). These antibiotic-kill experiments were performed in 6-well polystyrene plates (Celltreat Scientific Products).

### Viable cell density.

The number of viable bacteria per ml, the cell density, was estimated from the number of CFU by serial dilution in 0.85% saline and plating on LB (1.6%) agar plates.

### Antibiotics and their sources.

The following antibiotics were used in these experiments: ampicillin, chloramphenicol, colistin, gentamicin, kanamycin, oxacillin, tetracycline, and vancomycin (Sigma); azithromycin and tobramycin (Tocris); daptomycin (TCI); erythromycin (MP Biochemicals); ciprofloxacin and rifampin (Applichem); meropenem (Combi-Blocks), linezolid (Chem Impex, Int.).

### MICs.

*For* both S. aureus Newman and E. coli MG1655, antibiotic MICs were estimated using a 2-fold microdilution procedure (77). Two different initial concentrations of antibiotics were used to obtain accurate measurements from the 2-fold micro dilution procedure. The estimated MICs of each of the antibiotic bacteria combination are listed in Table 1 .

**TABLE 1:** Antibiotic MICs for S. aureus and E. coli strains

Antibiotic (abbreviation)	Antibiotic MIC (μg/ml)		
	S. aureus Newman	E. coli MG1655	E. coli MG1655 (*hipA7* mutant)
Ampicillin (AMP)	4.7	4.7	4.7
Azithromycin (AZM)	2.3	19.0	38.0
Chloramphenicol (CAM)	13	4.7	4.7
Ciprofloxacin (CIP)	0.2	0.6	0.6
Colistin (CST)	NA^*a*^	1.1	1.1
Daptomycin (DAP)	1.6	NA	NA
Erythromycin (ERY)	0.6	NA	NA
Gentamicin (GEN)	0.8	9.3	9.3
Kanamycin (KAN)	2.3	4.7	4.7
Linezolid (LZD)	1.1	NA	NA
Meropenem (MEM)	NA	1.2	0.6
Oxacillin (OXA)	0.3	NA	NA
Rifampin (RIF)	0.002	9.4	9.4
Tetracycline (TET)	0.6	2.3	75.0
Tobramycin (TOB)	0.3	1.2	2.3
Vancomycin (VAN)	1.6	NA	NA

a NA, not applicable.

### N(24)/N(0) ratios.

As our measure of the efficacy of the different antibiotics to killing the exposed bacteria, we used the ratio of the viable cell density after 24 h of exposure to the drug to the initial density estimated prior to exposure, i.e., N(24)/N(0). For each experiment, we estimated the N(0) and N(24) densities (in CFU) with three independent serial dilutions. For each antibiotic-bacterium combination, unless otherwise stated, we ran at least three independent experiments and calculated the means and standard errors of the N(24)/N(0) ratios.

### Antibiotic-mediated killing of exponentially growing bacteria.

For these experiments, overnight cultures of S. aureus and E. coli MG1655 were added to broth at a ratio of 1:100, followed by incubation for 1.5 h, and the density of the cultures, N(0), was estimated. Next, 5-ml samples of these cultures were put in 6-well plates, and antibiotics were added. The cultures with the antibiotics were incubated for 24 h, at which time the viable cell densities [N(24)] were estimated.

### Antibiotic-mediated killing of stationary-phase bacteria.

For the stationary-phase experiments with both S. aureus and E. coli, we used cultures that had been incubated under optimal growth conditions for 48 h. At 48 h, the viable cell densities of the stationary-phase cultures [N(0)] were estimated. Then, 5-ml aliquots were put in 6-well plates, the antibiotics were added, and the cultures were incubated with shaking for another 24 h; the viable cell densities were then estimated.

### Antibiotic-mediated killing of *S. aureus* bactericidal persisters.

Overnight MHII cultures of S. aureus Newman were diluted 1/10 in fresh MHII, and 25× MIC ampicillin was added immediately. After 24 h, the viable cell densities [N(0)] were estimated. Then, 5-ml samples of these ampicillin-treated cultures were put in 6-well plates, the second antibiotic(s) was added, and the cultures were incubated with shaking for another 24 h.

### Antibiotic-mediated killing of *E. coli hipA7* bactericidal persisters.

Exponentially growing LB cultures of E. coli *hipA7* were exposed to 10× MIC ciprofloxacin or ampicillin for 4 h [N(0)] and then treated with the second antibiotic for another 24 h [N(24)].

### Antibiotic-mediated killing of antibiotic-induced static populations.

Antibiotic-induced static populations were generated by exposing exponentially growing S. aureus and E. coli to bacteriostatic drugs for 24 h, and the viable cell density [N(0)] was estimated. The culture was divided into 5-ml aliquots in 6-well plates, and the second antibiotic was added. The cultures were maintained for another 24 h, and the viable cell densities [N(24)] were estimated.

### A caveat.

The magnitude of the variation in the extent of antibiotic-mediated killing and the levels of persistence among independent replicas was often substantial. We did a great deal of replication and are confident regarding the results reported here in a semiquantitative way, meaning that we are convinced that the relative extents of killing and the levels of persistence by the antibiotics used here would be obtained in parallel experiments in other laboratories. On the other hand, we would be surprised if the absolute numbers of bacteria surviving exposure to these drugs in these experiments would be identical to those reported here.

Curiously, in the persistence literature, this variation is rarely reported; for an exception, see the study by Johnson and Levin (78). Contributing to this variation are, of course, differences among batches of media and particularly broths. Moreover and perhaps more important is the variation in the strains used by different laboratories and possibly even by the same laboratory at different times. Although designated E. coli MG1655 or S. aureus Newman, every time these bacteria are recloned, there is a possibility of random fixation mutations, which over time can affect the phenotype of the cell line, including declines in fitness, a phenomenon referred to as Muller’s Ratchet (79). This accumulation of mutations can be seen in the DNA sequence data, as well as in phenotypic variation among isolates of strains with the same name (see, for example, references 80 and 81). In addition, it seems clear that persistence and the response of bacteria to antibiotics are multicausal phenotypes; as is the rule in complex systems, the quantitative reproducibility of experiments is frequently impaired, since small and difficult-to-control variations in the initial variables can quantitatively influence the final result (82).
